# Supplementary material for: Anticancer Effect of Heparin–Taurocholate Conjugate on Orthotopically Induced Exocrine and Endocrine Pancreatic Cancer
Source: Cancers (Basel). 2021 Nov 18;13(22):5775. doi: 10.3390/cancers13225775 (PMC8616444; doi:10.3390/cancers13225775)
Supplement: Supplementary file 1 [file cancers-13-05775-s001.zip › cancers-1438205-supplementary.pdf]

Supplementary Information

Anti-cancer effect of heparin taurocholate conjugate on orthotopically induced exocrine and endocrine pancreatic cancer

Hae Hyun Hwang[1,*], Hee Jeong Jeong[1,*], Sangwu Yun[1,*], Youngro Byun[2], Teruo Okano[3], Sung Wan Kim[4] and Dong Yun Lee[1,3,4**]

Supporting figure: S1~S6

ABBREVIATIONS

PDAC, pancreatic ductal adenocarcinoma; PNET, pancreatic neuroendocrine tumor; VEGF, Vascular endothelial growth factor; RTKs, receptor tyrosine kinases; VEGFR, Vascular endothelial growth factor receptor; HIT, heparin-induced thrombocytopenia; LHT, Low molecular weight Heparin-Taurocholate conjugate; LMWH, low molecular weight heparin; N-hydroxysuccinimide, NHS; 1-ethyl-3-(3-dimethylaminopropyl) carbodiimidehydrochloride, EDAC; HUVECs, Human umbilical vein endothelial cells; EGM, endothelial growth medium; CCK-8, Cell Counting Kit-8; DAPI, 4′,6-diamidino-2-phenylindole; GFR, growth factor-reduced; OCT, optimum cutting temperature; ERK, extracellular signal-regulated kinase; FAK, focal adhesion kinase.

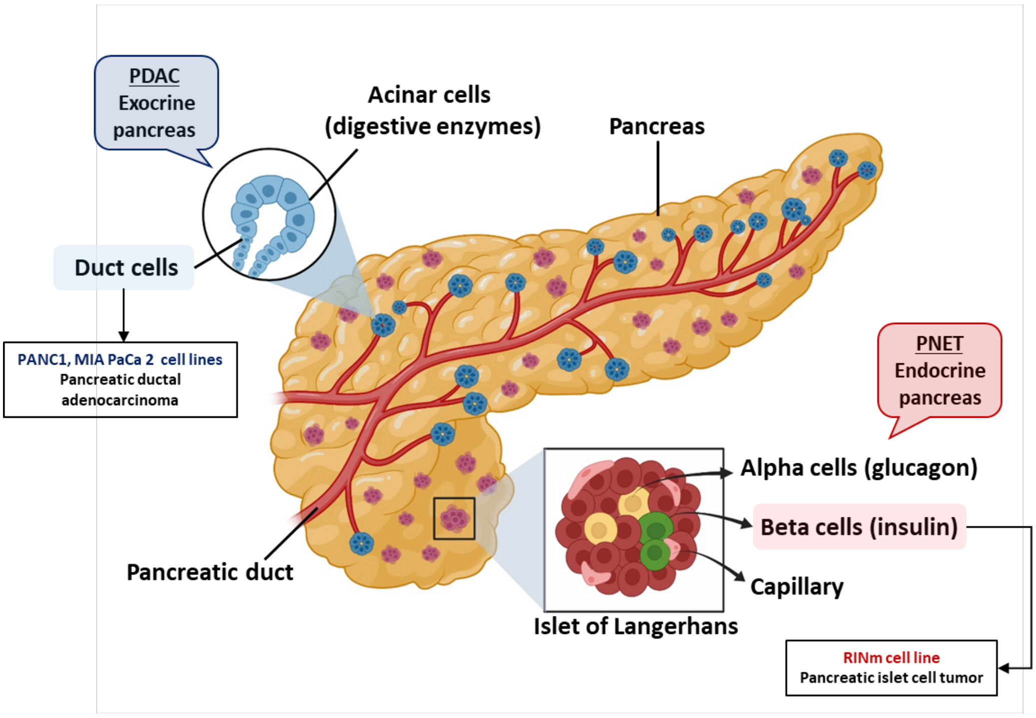

Figure S1. Origin of pancreatic tumor cell lines. PANC1 and MIA PaCa-2 are pancreatic ductal adenocarcinoma (PDAC) cell lines from the duct of exocrine pancreatic tumor. RINm is a rat insulinoma cell line from beta cells of pancreatic neuroendocrine islet cell tumor (PNET).

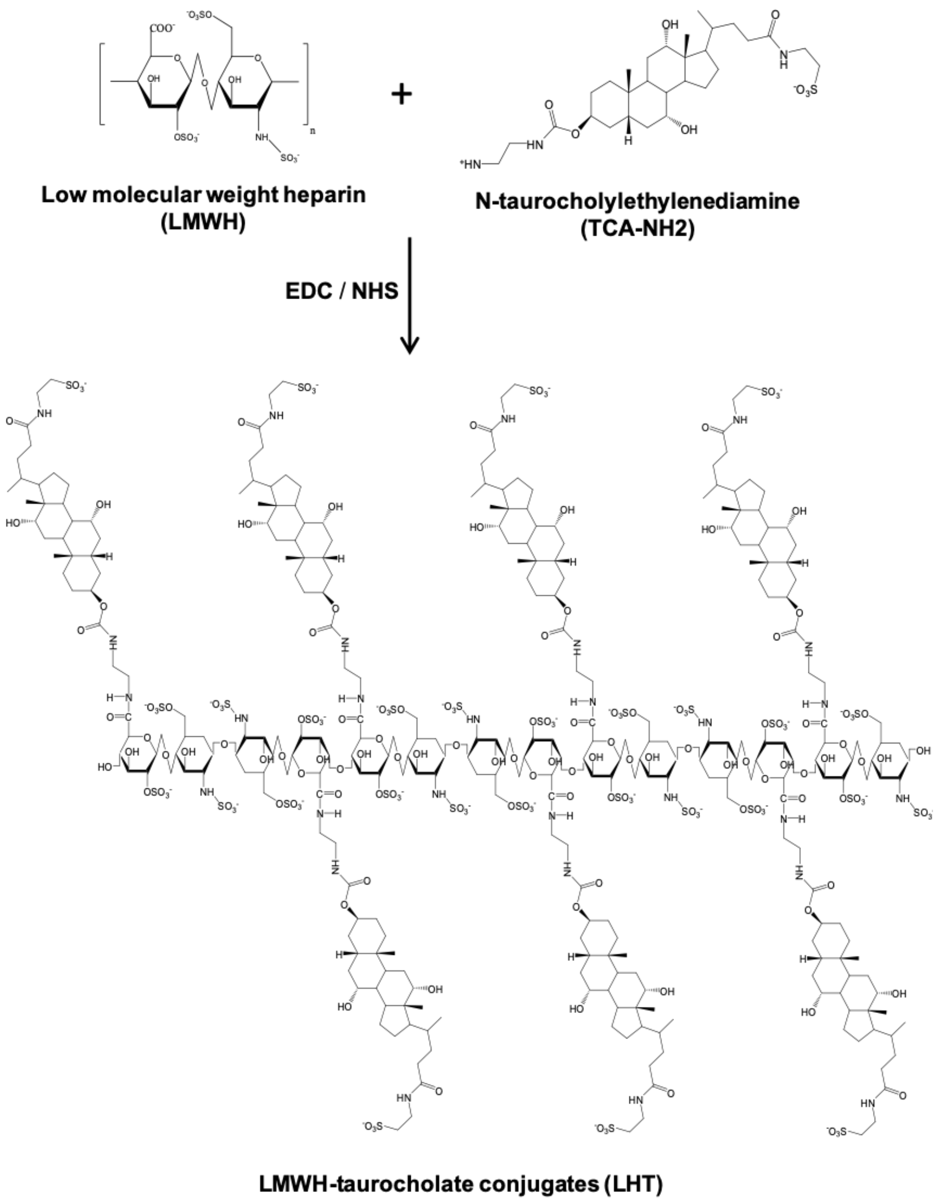

Figure S2. Chemical scheme for preparation of low-molecular weight heparin-taurocholate (LHT). Low molecular weight heparin (LMWH) was chemically conjugated with taurocholate modified with ethylenediamine.

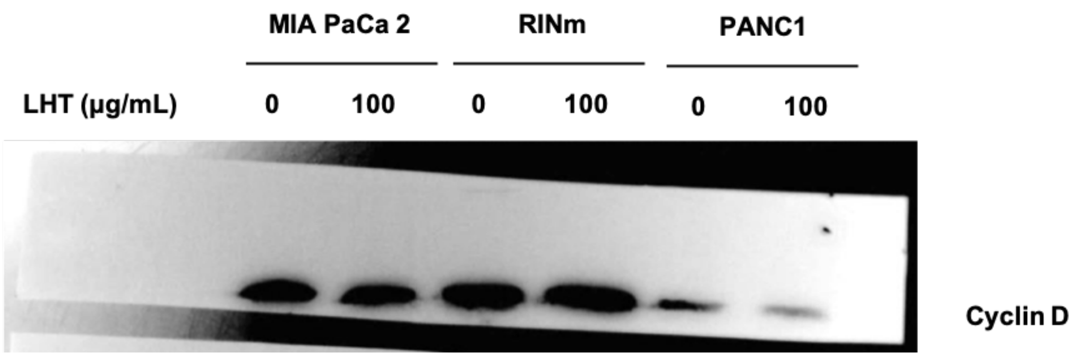

Figure S3. Western blot (Full-length blot) of cyclin D molecule in the cell lysates of pancreatic cancer cells without or with LHT (100 µg/mL).

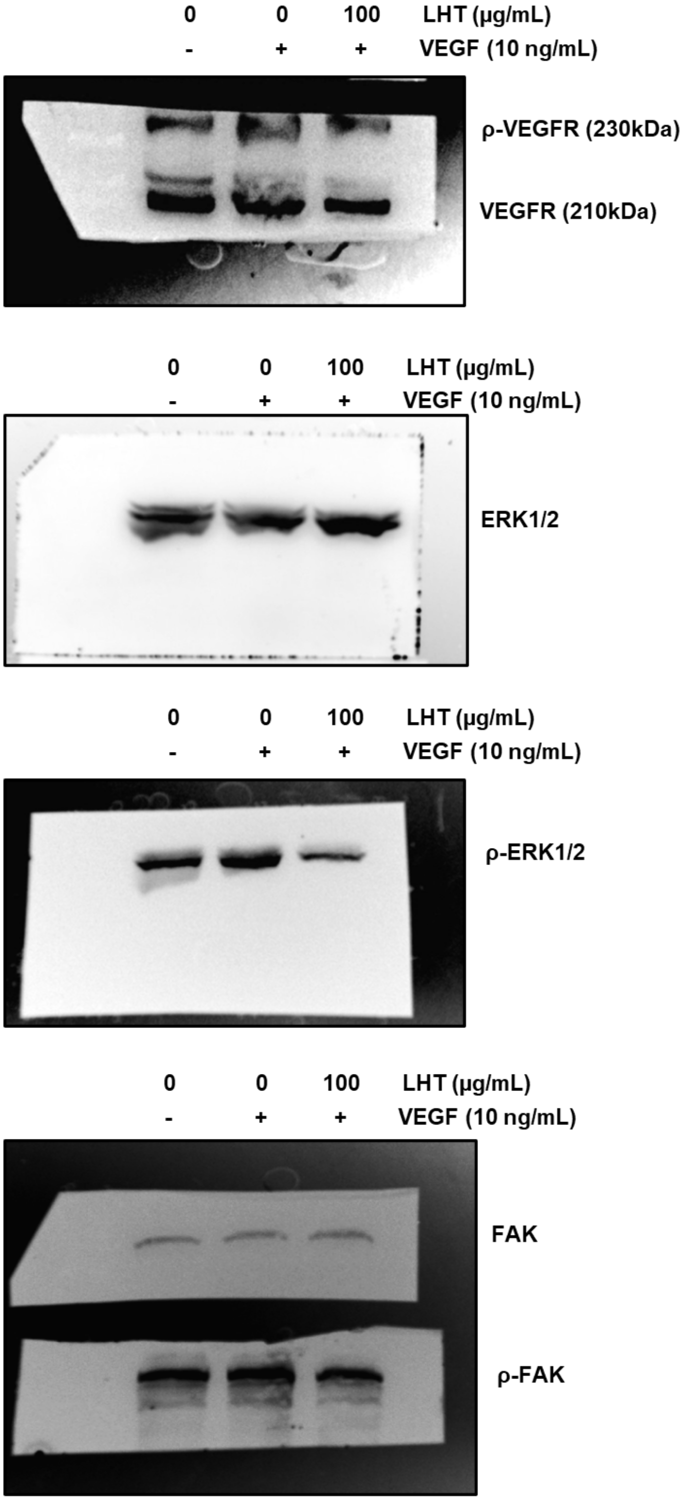

Figure S4. Western blot (Full-length blots) of cell lysate of HUVECs after cultivation with VEGF (10 ng/mL) or VEGF with LHT (100 μg/mL) for 24 h. The cropped blot images were presented in **Figure 6**.

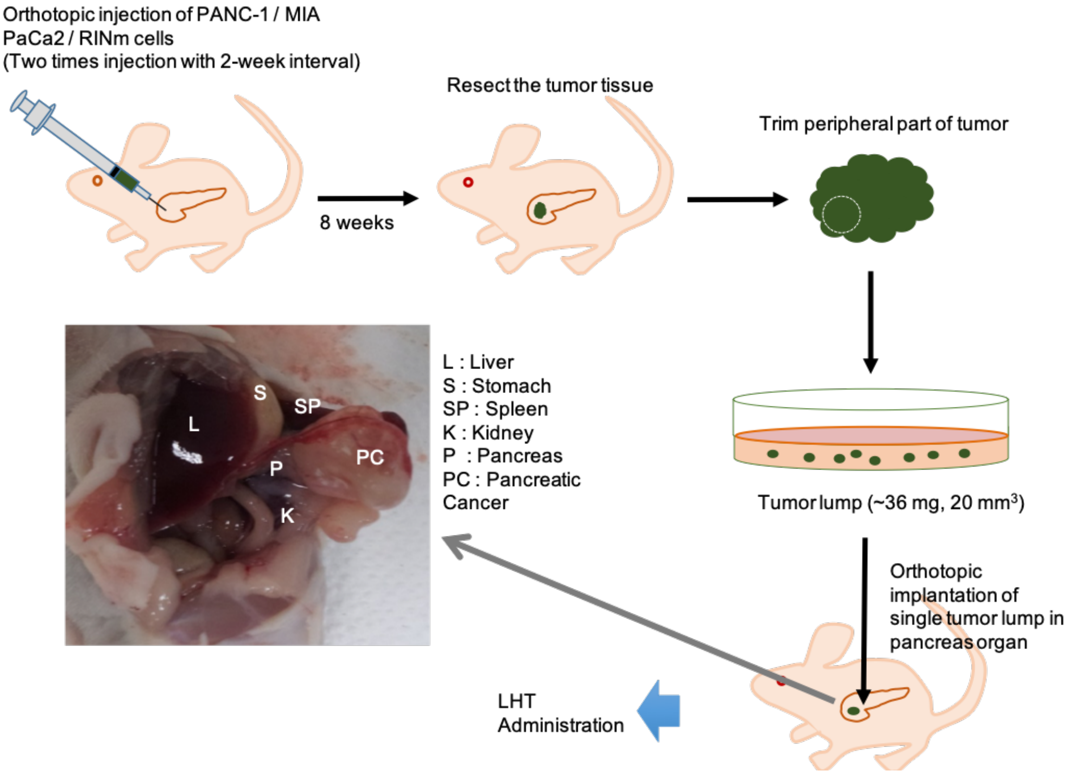

Figure S5. Surgical protocol to prepare three different kinds of orthotopic pancreatic cancer models using PANC-1, MIA PaCa-2 and RINm cells.

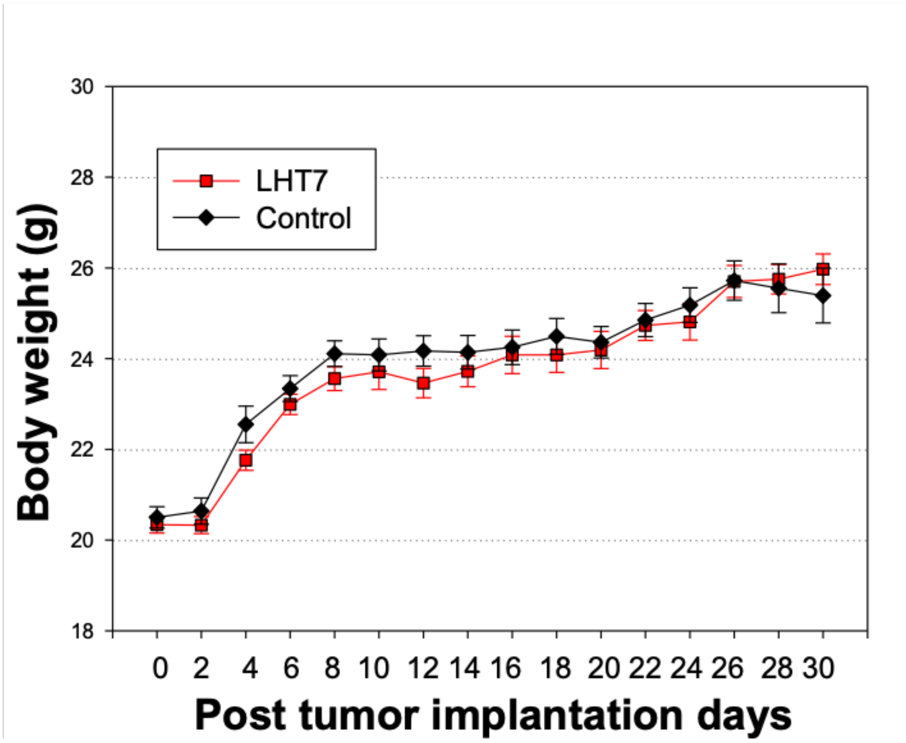

Figure S6. Body weight of orthotopic pancreatic mouse after intravenous administration of LHT (5 mg/kg/once every 2 days) or PBS vehicle for 30 days. Data were expressed with mean ± s.e.m. (n=5).
